# Multiple ileal perforations due to regular diclofenac sodium injections: a case report

**DOI:** 10.1186/1756-0500-6-129

**Published:** 2013-04-02

**Authors:** Won Seok Park, Sang Wook Kim, Seok Lee, Soo Teik Lee, Ho Sung Park

**Affiliations:** 1 Division of Gastroenterology and Hepatology, Departments of Internal Medicine, Daejon St. Mary’s Hospital, The Catholic University of Korea, Daejon, Republic of Korea; 2 Aerospace Medical Center, Republic of Korea Air Force, Cheongwon-gun, Chungcheongbuk-do, Republic of Korea; 3 Departments of Internal Medicine and Research Institute for Medical Science, Chonbuk National University College of Medicine, Geumam 2-dong, Deokjin-gu, Jeonju, 561-712, Republic of Korea; 4 Departments of Pathology, Chonbuk National University College of Medicine, Jeonju, Republic of Korea

**Keywords:** Diclofenac sodium, Ileum, Intestinal perforation, Non-steroidal anti-inflammatory agents

## Abstract

**Background:**

Although the adverse effects of non-steroidal anti-inflammatory drugs (NSAIDs) on the upper gastrointestinal tract have been well characterized, those specific to the lower gastrointestinal tract are less clear, as only a limited number of articles and case reports exist in the literature.

**Case presentation:**

We describe a case of a 69-year-old woman who presented to the emergency department due to sudden onset abdominal distension and pain. Notably, she reported using intramuscular diclofenac sodium twice daily for 14 days after knee joint replacement surgery. The patient denied any other coexisting diseases. As a subsequent X-ray and computed tomography (CT) scan showed free air in the abdomen, an exploratory laparotomy was performed, revealing four free perforations in the terminal ileum. Findings on microscopic analysis were non-specific.

**Conclusion:**

We report a unique case of multiple ileal perforations due to regular diclofenac sodium injections and contend that ileal perforation can be considered as a source for pneumoperitoneum with concomitant peritonitis in patients with a history of NSAID use if other possibilities are excluded.

## Background

Non-steroidal anti-inflammatory drugs (NSAIDs) – including aspirin – have a long history of clinical use given their potent antipyretic, analgesic, and anti-inflammatory effects. However, NSAIDs also have a well-described serious side effect profile, most notably including gastrointestinal injury. With 7.9% of the Korean population considered elderly [1], NSAID use continues to increase, with NSAID-related gastrointestinal injury now a common clinical problem [2].

As capsule endoscopy and balloon enteroscopy are now able to detect even the smallest of small intestinal lesions, NSAID-induced enteropathy has become a topic of great interest in the gastroenterology literature. Moreover, new data now suggest that the prevalence of NSAID-induced enteropathy is higher than previously expected, and is likely continuing to increase [3]. Although the gross appearance of NSAID-induced enteropathy can vary significantly – including diaphragm-like strictures, ulcers, erosions, and mucosal redness – few case reports of NSAID-induced ileal perforation exist. Herein, we report a case of multiple ileal perforations secondary to diclofenac use.

## Case presentation

A 69-year-old woman presented to the emergency department with sudden onset abdominal distension and mild abdominal pain though denied diarrhea or fever. Notably, she reported that she had been using intramuscular diclofenac sodium twice a day for the past 14 days due to pain stemming from a recent knee joint replacement surgery. The patient denied any other coexisting diseases.

Physical examination revealed a distended abdomen with slight tenderness in the right lower quadrant. Although all laboratory tests – including the Widal test – were normal, a simple chest X-ray showed free air in the abdomen. A computed tomography scan of the abdomen was then performed, showing a large fluid collection and pneumoperitoneum, though no definite mass or site of perforation could be identified.

**Figure 1:**
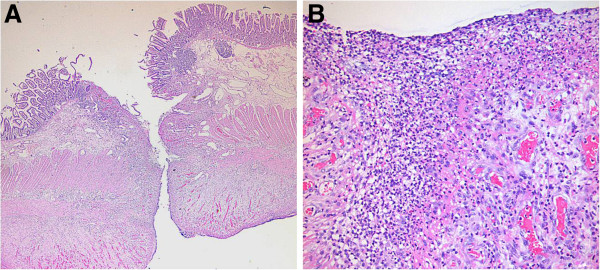
**Histopathology showing nonspecific inflammation and excluding IBD, vasculitis, and vascular thrombi.** The low power view (**A**) reveals an ulcer with perforation (H&E, x20). The high power view (**B**) reveals necrotic debris intermixed with inflammatory cells (predominantly neutrophils) and granulation tissue formation at the perforated ileal wall (H&E, x200).

Consequently, the patient underwent an emergent laparotomy, revealing multiple mucosal defects in the terminal ileum, including a total of four discrete perforations, each approximately 5 mm in size. However, a subsequent microscopic analysis did not reveal any specific findings around the sites of perforation, such as inflammatory cell infiltrates with thrombi, malignancies, or findings suggestive of inflammatory bowel disease (Figure 1). Accordingly, a diagnosis of multiple ileal perforations secondary to diclofenac use was reached, as the patient had no specific past medical history and the relevant laboratory testing and histopathology did not suggest any other underlying etiology. After surgery, the patient had an unremarkable course of recovery, and was followed as an outpatient without any additional complications.

## Discussion

NSAIDs have a well-characterized adverse event profile, including many upper gastrointestinal tract-related complications. These side effects clearly contribute significantly to the morbidity and mortality of individuals treated with this class of medications. However, NSAIDs are also capable of producing similar complications in the lower gastrointestinal tract, including the jejunum and ileum, and it is generally well accepted that NSAID-induced gastrointestinal injury occurs more frequently in the small bowel than in the stomach [4].

Though the incidence of NSAID-induced enteropathy is believed to be higher than previously expected, the exact rate has never been determined. According to one study including the post-mortem results of 713 patients both with and without a history of NSAID use, nonspecific small-intestinal ulcerations were found in 21 (8.4%) of NSAID users and 3 (0.6%) nonusers. Additionally, three long-term NSAID users were found to have died from complications arising from perforated nonspecific small-intestinal ulcers [5]. Another similar study also reported that after enteroscopy, jejunal or ileal ulcerations were detected in 47% of patients treated with NSAIDs for rheumatoid arthritis [6].

Prostaglandin is critically involved in regulating the gastrointestinal blood flow as well as other various mucosal functions. As such, the NSAID-induced decrease in prostaglandin production is believed to represent the primary cause of small bowel injuries due to NSAID use. Specifically, NSAIDs decrease endogenous mucosal prostaglandins, which results in decreased amounts of intestinal mucus, other microcirculatory disturbances secondary to pharmacologically increased intestinal motility, intercellular junction disruption, and increased mucosal permeability. The subsequent mucosal injuries can occur secondary to damage caused by bile acid, proteolytic enzymes, intestinal bacteria, or other toxins. Moreover, all of these processes induce inflammatory cytokine production as well as neutrophil infiltration, with the lipopolysaccharide/toll-like receptor 4 pathway also playing an important role in the etiology of such mucosal injuries [7].

The most basic principle in the treatment of NSAID-induced injuries is to discontinue all NSAID use. However, often only a temporary cessation of NSAID use is possible, as many patients require NSAIDs daily for chronic pain or as an anti-platelet agent. Although some preliminary evidence suggests that misoprostol, metronidazole, and/or sulfasalazine have some effect in treating NSAID-induced enteropathy, no confirmatory data exist. In fact, one recent double-blind, randomized controlled trial showed that neither metronidazole nor misoprostol was able to significantly reduce the increased mucosal permeability secondary to indomethacin after one week [8]. Another similar study of rheumatoid arthritis patients treated with NSAIDs found that sulfasalazine reduced both intestinal inflammation and blood loss, while all other disease-modifying antirheumatic drugs (DMARDs) did not [9]. Furthermore, while selective COX-2 inhibitors have been shown to decrease the rate of complications in the upper gastrointestinal tract, it remains unclear if these agents are similarly effective in preventing NSAID-related complications in the small bowel [10].

In the case described here, the patient received regular twice-daily injections of diclofenac sodium for two weeks. Similarly, the subsequent laboratory and histopathologic findings were unable to identify any etiology for her ileal perforations other than NSAID-induced gastropathy, including inflammatory bowel disease, trauma, typhoid, tuberculosis and amoebiasis. Accordingly, a diagnosis of diclofenac sodium-induced multiple ileal perforation was reached.

## Conclusion

We report a unique case of multiple ileal perforations due to regular diclofenac sodium injections and contend that ileal perforation can be considered as a source for pneumoperitoneum with concomitant peritonitis in patients with a history of NSAID use if other possibilities are excluded.

## Consent

Written informed consent was obtained from the patient’s father for publication of this case report.

## Competing interests

The authors declare that they have no competing interests.

## Authors’ contributions

PWS and KSW took part in the design of the study, was involved in patient treatment and participated in writing the manuscript. LS was a major contributor in writing the manuscript. LST involved the treatment procedures. PHS performed the pathologic review. All authors read and approved the final manuscript.
